# Convergent Bacterial but Divergent Fungal Communities in the Tobacco Rhizosphere Under Intensive Management on Contrasting Soils

**DOI:** 10.3390/microorganisms14081849

**Published:** 2026-08-20

**Authors:** Shuang Peng, Dan Song, Beibei Zhou, Yiming Wang

**Affiliations:** 1 College of Environment and Ecology, Jiangsu Open University, Nanjing 210017, China; 2 State Key Laboratory of Soil and Sustainable Agriculture, Institute of Soil Science, Chinese Academy of Sciences, Nanjing 210008, China; 3 Jiangsu Collaborative Innovation Center for Solid Organic Waste Resource Utilization, Nanjing 210095, China

**Keywords:** tobacco, rhizosphere microbiome, clay loam soil, sandy loam soil, plant–microbe interactions

## Abstract

The rhizosphere microbiome is critical for plant health, yet how soil type and intensive management jointly govern its assembly remain unclear. Here, we hypothesized that soil type acts as a primary environmental filter, while intensive cultivation (plant growth plus fertilization) imposes additional selective pressures that differentially shape bacterial versus fungal communities. Using flue-cured tobacco (K326) grown in clay loam and sandy loam soils under field conditions, we examined the rhizosphere microbiome at the topping stage. Intensive cultivation significantly altered rhizosphere physicochemical properties. Key nutrients, including organic matter (OM), dissolved total nitrogen (DTN), available phosphorus (AP), and available potassium (AK), were markedly enriched. Rhizosphere soil pH exhibited a bidirectional shift relative to the corresponding bulk soil, converging to a narrow range (7.4–7.8) in both soil types. Root activity and fertilization imposed contrasting selective pressures on the two microbial kingdoms: bacterial diversity declined slightly, indicating strong deterministic selection, whereas fungal diversity increased, reflecting adaptation to root-generated niches. Differential abundance analysis identified 38 bacterial OTUs as a core rhizosphere-adapted microbiome shared across both soil types, demonstrating robust fitness in the nutrient-enriched rhizosphere environment under intensive management. No shared core fungal OTUs were detected, underscoring strong soil legacy effects and higher habitat specificity in fungi. Notably, the core bacterial microbiome was dominated by K-strategists (slow-growing, resource-efficient taxa) that exhibited opportunistic traits capable of rapidly exploiting nutrient pulses in the rhizosphere. Together, these findings reveal that soil type acts as a critical filter modulating plant–microbe interactions under intensive agriculture, while bacteria and fungi employ divergent ecological strategies in response to selection pressures. This work provides both theoretical and practical insights for optimizing tobacco cultivation and sustaining soil microecological health.

## 1. Introduction

Flue-cured tobacco (*Nicotiana tabacum* L.) is an important economic crop cultivated worldwide [1]. Its yield and quality depend not only on varietal genetic traits and agronomic management, but also on the health of the soil ecosystem [2]. The rhizosphere, a narrow zone of soil directly influenced by plant roots, represents a critical interface for plant–microbe interactions that significantly impact plant health, nutrient acquisition, and soil ecosystem functioning [3]. Plants actively shape their rhizosphere microbiome through the release of a complex blend of root exudates, including sugars, organic acids, and secondary metabolites, a process often described as the “rhizosphere effect” [4,5]. This selective recruitment leads to the formation of a microbial community that is distinct from the bulk soil, and a core microbiome that is consistently associated with a plant species across different environments [6,7]. This microbiome is known as the “second genome” of plants [8], playing an indispensable role in promoting nutrient absorption, synthesizing plant hormones, resisting pathogen invasion, and alleviating abiotic stress [9].

Tobacco is typically cultivated under intensive management, characterized by high planting densities and substantial fertilizer inputs. In tobacco cultivation, for example, farmers regularly apply fertilizer to get high yield and quality [10]. This creates a nutrient-rich area around the roots. However, how this strong anthropogenic selection pressure interacts with the natural selective forces exerted by plant roots to collectively structure rhizosphere microbial communities, particularly across soil matrices that differ markedly in their physicochemical properties, remains poorly understood. Soil serves as both a microbial reservoir and a physicochemical filter; its intrinsic properties (e.g., texture, pH, background nutrient levels) likely exert profound influences on the recruitment efficiency of rhizosphere microorganisms and the resulting community composition [11,12]. For example, red soil, characterized by high clay content, exhibits strong water and nutrient retention capacity, whereas sandy soil, with good aeration, is prone to nutrient leaching [13,14]. These two contrasting soil habitats therefore constitute an ideal model system for investigating the interplay between the soil legacy effect and the rhizosphere effect.

The assembly of the rhizosphere microbiome is thought to be governed by deterministic processes, such as host selection and environmental filtering, and stochastic processes [15]. However, existing research often faces challenges in analyzing this complex interaction. A key scientific question is as follows: under intensive agriculture, does the integrated selective pressure (combining root activity and fertilization) lead to a convergent core microbiome across different soil types, or does soil type act as a persistent filter that preserves distinct community structures [16,17] even within this strongly managed system? While root exudates are known to be powerful mediators, their ability to shape community structure may be limited or modified by the initial soil microbial diversity and physicochemical properties, such as pH, organic matter, and nutrient availability [18,19]. Most studies have focused on bacterial communities, yet fungi, with their different life-history strategies and often closer symbiotic relationships with plants, may respond differently to plant selection and soil filters [20]. This underscores the need for a kingdom-specific investigation of the factors driving rhizosphere assembly.

It remains unclear whether tobacco plants, when grown intensively in soils with starkly contrasting physicochemical properties, can assemble a consistent core rhizosphere microbiome. To address this question more rigorously, the present study does not attempt to artificially disentangle the effects of fertilization from those of root activity. Instead, we treat the “rhizosphere microenvironment formed under intensive cultivation” as an integrated selective system, and ask whether soil type can override the outcome of this integrated selection. Accordingly, this study aims to: (1) systematically characterize the changes in physicochemical properties from bulk soil (affected by fertilization) to rhizosphere soil of flue-cured tobacco cultivar K326 in both clay loam soil and sandy loam soil; (2) reveal the diversity, composition, and structural responses of bacterial and fungal communities along these gradients using high-throughput sequencing, with particular emphasis on kingdom-specific differences; and (3) identify the core rhizosphere-adapted microbiome shared across soil types and characterize its ecological strategies (e.g., r/K selection traits and niche breadth) to understand how soil type modulates the functional composition of the assembled microbial communities. We hypothesize that, despite the strong selective pressures imposed by intensive cultivation, soil type will act as a critical “filter” or “modulator” that determines the ultimate composition and ecological resilience of the rhizosphere microbiome. The conclusions of this study will provide a more realistic and robust framework for understanding the interactive effects of genotype (plant), environment (soil), and management (fertilization) on microbiomes in real-world agroecosystems.

## 2. Materials and Methods

### 2.1. Description of the Experiment

The experimental site is located in Zhangjia Village, Chengjiang County, Yuxi City, Yunnan Province, which has a mid-subtropical plateau monsoon climate. The experimental field covered an area of 0.2 hectares, where tobacco (variety K326) was cultivated in two distinct soil types that differed substantially in origin and texture. One soil (SL) was a sandy loam, consisting of 40% coarse sand, 21% fine sand, 17% silt, and 22% clay; it originated from recent fluvial deposits of a nearby river and had a bulk density of 1.25 Mg/m^3^, a saturated water content of 42.2%, a porosity of 52.8%, and an initial pH of 8.75. The other soil (CL) was a clay loam, containing 3% coarse sand, 10% fine sand, 32% silt, and 55% clay; it was derived from local red soil and had a bulk density of 1.1 Mg/m^3^, a saturated water content of 53.2%, a porosity of 58.5%, and an initial pH of 7.39. 

The experiment was arranged in a completely randomized block design with three blocks. Each block contained two soil-type treatments, with three replicate plots (20 m × 15 m per plot). Fertilizers were applied following local conventional practice for flue-cured tobacco cultivation. The total application rates were: specialized compound fertilizer (N:P_2_O_5_:K_2_O = 12:6:14) at 600 kg/ha, potassium sulfate (K_2_O = 50%) at 375 kg/ha, organic fertilizer (decomposed livestock manure) at 1500 kg/ha, and seedling-promoting fertilizer (water-soluble, N:P_2_O_5_:K_2_O = 28:0:5, commercial product “Xin Yunye”, manufactured by Yunnan Yunye Fertilizer Co., Ltd.,Kunming, China) at 45 kg/ha. The compound fertilizer was applied as a basal dressing at transplanting (128 kg/ha) and as a topdressing twice thereafter: the first topdressing (150 kg/ha) was applied 15–20 days after transplanting, after breaking the plastic mulch and covering with soil, and the second topdressing (322 kg/ha) was applied as a balanced fertilizer solution depending on plant vigor during the ridging and hilling stage. The seedling-promoting fertilizer (45 kg/ha) was dissolved in water and applied as a liquid supplement to promote early growth. The organic fertilizer (1500 kg/ha) was applied as a basal dressing in the planting hole before transplanting and covered with dry soil. Potassium sulfate was applied in two splits: 300 kg/ha as a dry ring application at the time of plastic mulch removal and ridging, and the remainder (75 kg/ha) during the vigorous growth stage depending on plant demand. All fertilizers were applied manually around the base of each plant, and irrigation was used to facilitate nutrient uptake.

### 2.2. Soil Collection and Preparation

At the tobacco topping stage, three tobacco plants were randomly selected from each plot for sampling. The loose soil attached to the roots was gently shaken off and collected as bulk soil samples (SLB and CLB). For the rhizosphere soil samples (SLR and CLR), the roots were then shaken more vigorously, and the soil that remained firmly attached to the root surface was carefully removed by hand. Rhizosphere soil was collected and subsampled from five random locations within the material; these subsamples were pooled to form one composite sample per plant, yielding three biological replicates per soil type. Additionally, unplanted control soils were collected from adjacent areas that did not receive fertilization to serve as a baseline. All samples were promptly transported to the laboratory in ice-filled coolers. The soil samples were freed from large debris such as roots and stones, and then divided into two portions: one portion was frozen at −80 °C for subsequent DNA extraction, and the other was air-dried at 22–30 °C. The air-dried soil was passed through a 2 mm or 0.1 mm sieve for the determination of soil chemical characteristics.

### 2.3. Analysis of Soil Chemical Characteristics

Soil chemical properties were determined following the standard methods described by Lu [21]. Total nitrogen (TN) was determined using the modified Kjeldahl method. Total phosphorus (TP) was extracted via H_2_SO_4_-HClO_4_ digestion and subsequently quantified by spectrophotometry. Total potassium (TK) was measured using the NaOH fusion-flame photometer method. Dissolved total nitrogen (DTN) in soil was extracted with 2 M KCl and analyzed using a continuous-flow analytical system (San++ System, Skalar, Breda, Holland). Available phosphorus (AP) was extracted with sodium bicarbonate and measured by the molybdenum blue colorimetric method (Olsen method). Available potassium (AK) was extracted with ammonium acetate and determined by flame photometry. Organic matter (OM) was analyzed using the potassium dichromate volumetric method. Soil pH was measured using a combination glass electrode pH meter (REX, Shanghai, China) in a 1:2.5 (soil:water) suspension. Electrical conductivity (EC) was determined with a conductivity meter (DDSJ-308A, Leici, Shanghai, China) in a 1:5 (soil:water) suspension.

### 2.4. Soil DNA Extraction and Illumina MiSeq DNA Sequencing

Total genomic DNA of the microbial community was extracted using the FastDNA^®^ SPIN Kit (MP Biomedicals, Irvine, CA, USA), following manufacturer protocols. DNA concentration and purity were measured with an ND-1000 spectrophotometer (Thermo Fisher Scientific, Waltham, MA, USA), with DNA stored at −20 °C.

The microbial communities in soil samples were analyzed using the high-throughput sequencing method, conducted on an Illumina NovaSeq6000 platform (Novegene, Beijing, China). The V3-V4 region of the bacterial 16S rRNA gene was amplified with universal primers 341F/805R [22]. Fungal ITS1 regions were amplified with primers ITS5-1737F and ITS2-2043R [23]. The obtained sequences were subjected to quality control using Cutadapt (v1.9.1). High-quality sequences were clustered using Uparse (v7.0.1001) [24], where sequences with >97% similarity were grouped into the same operational taxonomic unit (OTU). For taxonomic assignment of bacterial OTUs, the representative sequences were classified using the Mothur software (v1.43.0) against the SILVA 132 SSU rRNA database [25], with a bootstrap cutoff of 0.8–1.0. For fungal OTUs, the representative sequences were classified using the BLAST (v2.2.22) method implemented in QIIME (v1.9.1) against the UNITE database (v7.2) [26]. Alpha diversity indices, including Shannon, Simpson, Chao1, and ACE, were calculated with QIIME (v1.9.1). The sequences were submitted to the NCBI Sequence Read Archive (SRA) database (accession no.: PRJNA1481388).

### 2.5. Statistical Analysis

The Bray–Curtis distance matrix was calculated and the β-diversity of the community was analyzed by nonmetric multidimensional scaling (NMDS). Permutational multivariate analyses of variance (PERMANOVA) were used to examine the difference in community structure. The R package DESeq2 was used to compare differences in the abundance of OTUs between the rhizosphere soil and the bulk soil in R software (v4.4.2). Only OTUs with *p*  <  0.05 (FDR-adjusted) and log2 (fold change) > 1 or <−1 were regarded as significantly different. Those OTUs enriched in the rhizosphere soil of the clay loam (CLR) and sandy loam (SLR) soils were defined as the sensitive taxa, and those enriched in both SLR and CLR were defined as the core taxa. Key species were further identified based on Zi and Pi values, representing within-module and among-module connectivity, respectively, following the method of [27]. The ribosomal RNA gene operon (rrn) copy number in bacterial genomes is a phylogenetically conserved trait that correlates positively with maximum potential growth rate and negatively with nutrient-use efficiency [28]. In general, microorganisms with a high rrn copy number tend to be r-strategists, while those with a lower rrn copy number tend to be K-strategists [29,30], characterized by slower growth but higher resource-use efficiency, which makes them better adapted to oligotrophic or stable environments [31]. The rrn copy numbers for bacterial OTUs were estimated based on the rrnDB database [32,33]. Niche width indices were calculated using the Levins model with the “spaa” package of R [34].

## 3. Results and Discussion

### 3.1. Response of and Variation in Soil Physicochemical Properties in the Rhizosphere

Soil physicochemical properties form the basis for microbial community structure and function. Tobacco cultivation, combined with ring fertilization, led to a significant enrichment of OM, DTN, AP, AK, and TN in the rhizosphere of both soil types (Table 1), indicating the influence of the “rhizosphere effect”. The significant nutrient enrichment in the rhizosphere is a direct result of the interactions between the plant, soil, and applied fertilizer. The ring-fertilized band provides the initial nutrient source. More importantly, tobacco roots secrete substantial photosynthates (e.g., mucilage, organic acids, sugars), which supply abundant carbon and energy to rhizosphere microorganisms, thereby significantly stimulating microbial activity [35]. These active microbes accelerate the decomposition of soil organic matter and nutrient mineralization. Concurrently, the root exudates themselves directly contribute to the soil organic matter pool [36]. Ultimately, this root exudate-driven “rhizosphere priming effect” [37] and fertilizer input jointly result in significant nutrient enrichment within the rhizosphere zone. The rhizosphere of the clay loam soil showed a greater improvement in nutrient metrics compared to the sandy loam soil (Table 1), likely due to its inherent properties. In contrast, the weaker nutrient and water retention capacity of sandy loam soil may lead to greater nutrient leaching, potentially attenuating the rhizosphere enrichment effect. This result highlights the importance of native soil properties in modulating the intensity of plant–soil interactions.

Soil pH is one of the most important factors affecting microbial community structure and elemental biogeochemical cycling. Tobacco planting and fertilization regulated the rhizosphere pH of two originally distinct soils towards a neutral range (7.4–7.8) (Table 1). This is not a passive process but rather an active “pH homeostasis” strategy employed by the plant [38]. Plants can alter the rhizosphere pH balance by secreting specific combinations of organic acids, protons, and/or enzymes like carbonic anhydrase. This precise regulation may have important ecophysiological significance: a neutral-pH environment helps increase the availability of sparingly soluble nutrients like phosphorus and optimizes the activity of most soil enzymes and microbes, thereby creating a more favorable rhizosphere microenvironment for nutrient acquisition [39]. Beyond nutrient availability, the observed pH convergence toward neutrality likely influences metal ion speciation and bioavailability. In the sandy loam soil, where the initial pH was alkaline (8.75), the substantial pH decrease in the rhizosphere zone (7.83) may enhance the availability of micronutrients such as iron (Fe), zinc (Zn), and copper (Cu), which are often poorly soluble under alkaline conditions [40]. These pH-driven changes in metal speciation could contribute to the observed shifts in microbial community composition, as different microbial taxa exhibit varying tolerances and requirements for metal ions [41]. Future studies incorporating metal fractionation analysis would help clarify the specific role of metal speciation in rhizosphere microbiome assembly under intensive management. Soil electrical conductivity (EC) also increased markedly in the rhizosphere of both soil types (Table 1), reflecting the accumulation of soluble salts from fertilizer application. This pH convergence, together with nutrient and salt enrichment, establishes a strongly selective microenvironment that differs fundamentally from the original soil and bulk soil, providing a physicochemical basis for subsequent microbial community shifts.

### 3.2. Variation in Microbial Community Diversity

After quality filtering, the average number of valid reads per sample was 66,322 (range: 40,447–84,759) for bacterial 16S rRNA gene amplicons and 76,159 (range: 51,774–80,338) for fungal ITS1 amplicons. Alpha diversity analysis revealed contrasting responses of bacteria and fungi to intensive planting (Table 2). Soils from the sandy loam group (SL, SLB, SLR) generally showed higher bacterial diversity and richness compared to clay loam soils (CL, CLB, CLR). Sandy loam soils, with their higher drainage and greater textural heterogeneity, may provide more spatially heterogeneous microhabitats. Soil textural heterogeneity has been shown to positively influence bacterial diversity [42], and sandy loam soils have been observed to harbor higher bacterial diversity than clay soils in certain agroecosystems [43]. However, the relationship between soil texture and bacterial diversity is context-dependent and can vary across ecosystems [44]. Notably, in both soil types, bulk soil samples (SLB, CLB) showed improved bacterial diversity and richness compared to their respective original soils (CL, SL), suggesting that nutrient input promoted the proliferation of various bacteria. However, compared with bulk soil, the bacterial diversity and richness indices decreased in rhizosphere soil (*p* > 0.05) (Table 2), indicating that nutrient and tobacco root exudates exert a slight selective effect on the proliferation of several bacterial communities.

**Figure 1:**
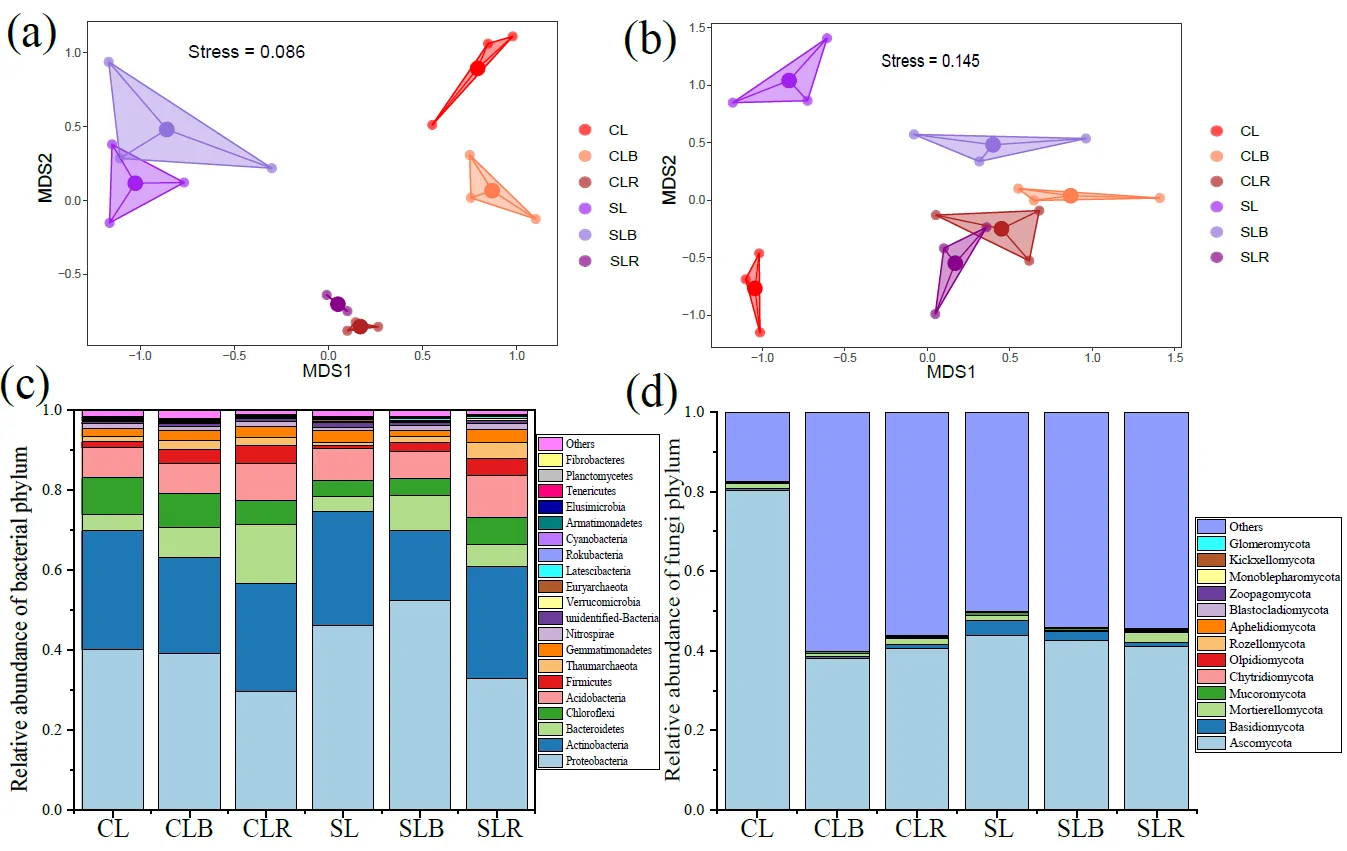
Nonmetric multidimensional scaling analysis of bacterial (**a**) and fungal (**b**) community structure in different soils. Relative abundance in the dominant bacterial (**c**) and fungal (**d**) phyla. Abbreviations: CL, clay loam soil; SL, sandy loam soil; B, bulk soil; R, rhizosphere soil.

**Figure 2:**
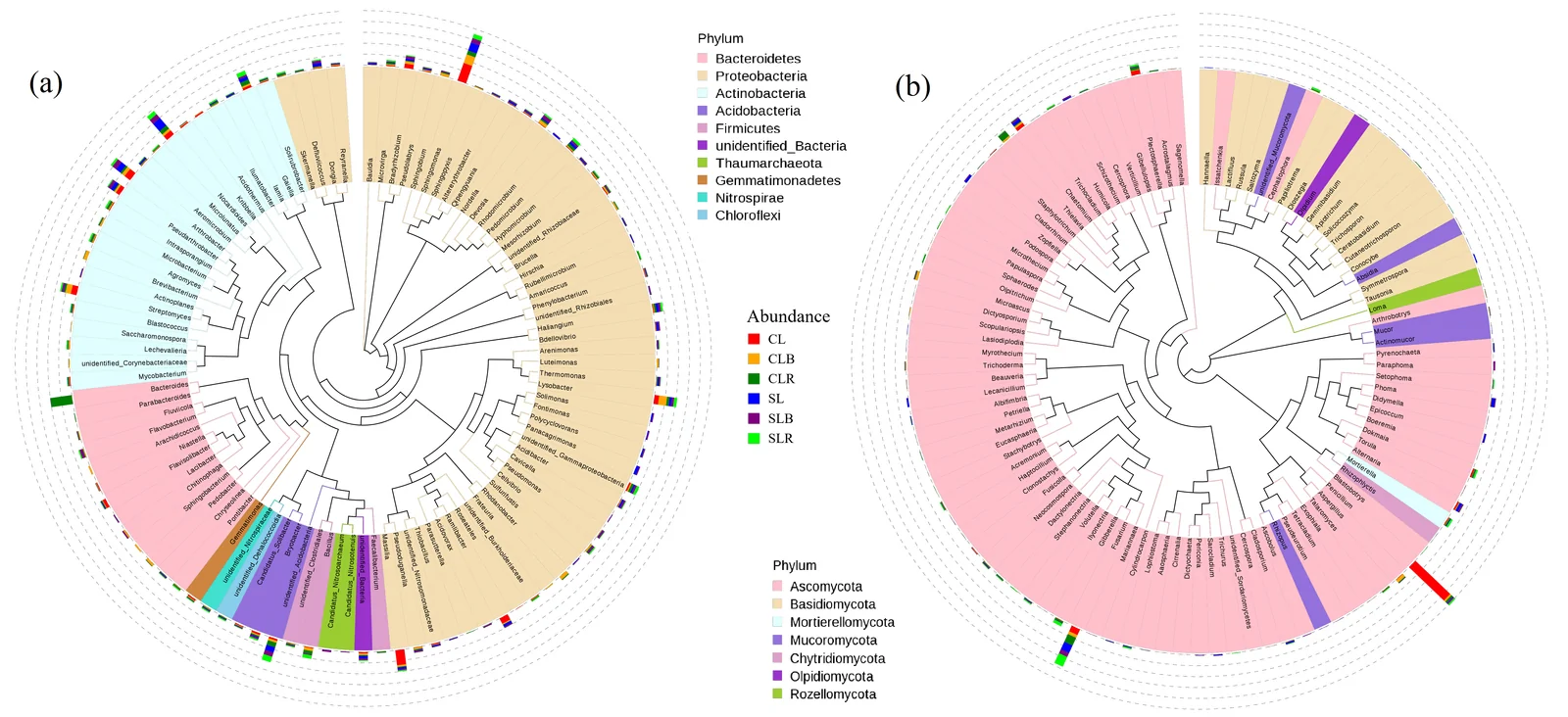
Phylogenetic tree of the top 100 bacterial (**a**) and fungal (**b**) genera. Abbreviations: CL, clay loam soil; SL, sandy loam soil; B, bulk soil; R, rhizosphere soil.

**Figure 3:**
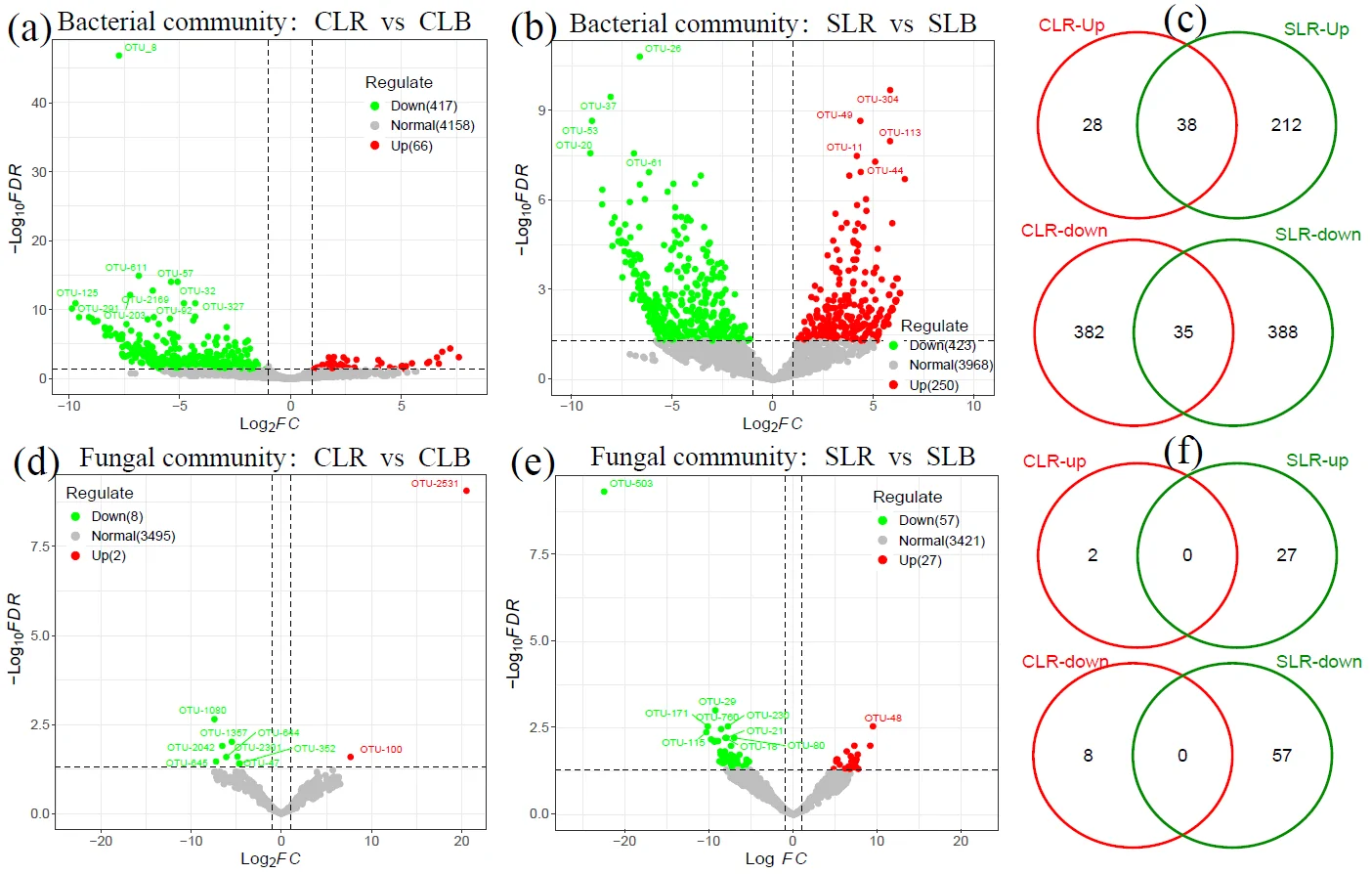
Volcano plots (**a**,**b**,**d**,**e**) showing the significantly enriched or depleted OTUs in the rhizosphere of each soil compared with bulk soil. Red dots indicate significantly upregulated OTUs, green dots indicate significantly downregulated OTUs, and gray dots represent OTUs with no significant difference (DESeq2, *p* < 0.05, FDR adjustment). Venn diagram of enriched or depleted bacterial (**c**) and fungal (**f**) OTUs in tobacco rhizosphere between clay loam and sandy loam soils. FC, fold change. Abbreviations: CL, clay loam soil; SL, sandy loam soil; B, bulk soil; R, rhizosphere soil.

Interactions between plant roots and soil microorganisms are critical for plant fitness in natural environments. In this study, bacterial diversity and richness were significantly different between the two soil types (Table 2). However, after cultivating the same tobacco variety, the overall structure and diversity of their rhizosphere microbiomes became similar (Figure 1 and Figure 2). This strongly suggests that intensive tobacco planting acts as a significant force in shaping its rhizosphere microbial community. The bacterial Venn diagram (a) shows a substantially larger number of shared OTUs among bacteria compared to fungi (Figure S1). The tobacco plant selectively “recruits” preferred microorganisms, such as the 38 bacterial OTUs enriched in both CLR and SLR in this study (Figure 3), while suppressing certain other microbial taxa, like the 35 bacterial OTUs that were simultaneously reduced in both CLR and SLR (Figure 3). Functioning as a powerful “filter” or “engineer”, the tobacco plant largely overrides the inherent differences originally present in the two soil types. Plants release a diverse array of metabolites into the rhizosphere, such as carboxylic acids (e.g., malic and citric acid), flavonoids, and secondary metabolites, to create a specific chemical niche that attracts beneficial microbes [9,45]. These signals selectively recruit microbial taxa from distinct soil microbial pools that can respond to and utilize them, thereby assembling rhizosphere communities with similar functional profiles. Mechanistic studies indicate that local microbial colonization can induce systemic shifts in exudation, influencing both plant and microbial gene expression [46]. Several papers report that such targeted recruitment correlates with enhanced plant disease resistance [47], nutrient acquisition [48], and stress tolerance [49]. Collectively, plant root exudates play an active, chemically specific role in recruiting and shaping beneficial microbial communities [45].

Conversely, nutrient input in the bulk soil reduced the diversity and richness of the fungal community, while root exudates not only mitigated this change but also further enhanced the diversity and richness of fungi in the rhizosphere soil (Table 2). This may be because of high levels of inorganic nitrogen and phosphorus input in bulk soil favoring some fast-growing saprophytic fungi, leading to a decrease in overall community diversity [50,51]. The subsequent significant rebound and enhancement of fungal diversity in the rhizosphere (Table 2) suggest that root activity (e.g., secreting complex organic compounds) provides diverse carbon sources and microhabitats for fungi [52]. This likely favors the proliferation of fast-growing saprophytic fungi and other opportunistic taxa, effectively alleviating the negative effects of fertilization and promoting the formation of a more diverse fungal community. It is important to note that the potential impact of these changes on the bulk soil microbiome, whether mediated by microbial dispersal or altered nutrient gradients, remains to be investigated.

### 3.3. Microbial Community Composition: Dominant Groups and Soil Specificity

At the phylum level, bacterial communities in both soils were dominated by *Proteobacteria*, *Actinobacteria*, and *Acidobacteria*, while fungal communities were predominantly composed of *Ascomycota* (Figure 1c,d). Rhizosphere colonization induced significant shifts in major bacterial phyla (Figure 1), with *Proteobacteria* decreasing and several taxa (*Actinobacteria*, *Acidobacteria*, *Firmicutes*, *Gemmatimonadetes*) enriched. Root exudates from tobacco have been shown to enrich specific soil microbiota, with notable effects on taxa such as *Streptomyces*, *Hydrophilus*, *Roseobacter*, *Proteobacteria*, *Actinobacteria*, *Burkholderia*, and *Bradyrhizobium* [53,54]. Several studies also report the enrichment of *Bacillus*, particularly *Bacillus amyloliquefaciens*, which is attracted by flavonoids, phenylpropanoids, terpenoids, and defense-related hormones secreted by the roots of diseased tobacco plants [55]. For instance, *B. amyloliquefaciens* ZM9 has been identified as a core microbial taxon enriched by tobacco root exudates [56]. Beyond tobacco, studies in other systems, such as maize, have demonstrated that benzoxazinoid exudation can increase the abundance of *Methylophilaceae* bacteria [57], which in turn antagonize pathogenic Xanthomonadaceae species [58].

Although the overall structure and diversity of the rhizosphere microbial communities were similar between SLR and CLR, differences remained. Specifically, 212 bacterial OTUs were enriched exclusively in SLR, while 28 were unique to CLR. An even greater number of bacterial OTUs were significantly reduced in a soil-specific manner (Figure 3). This indicates that the rhizosphere bacteria in the two soils retained a degree of “differentiation” in their responses at the species level, reflecting soil specificity. Root exudates, which are organic compounds secreted by plant roots, vary in composition depending on plant species, developmental stage, and environmental conditions [53,59,60]. They serve as both signals and nutrients, selectively recruiting different microbial taxa. For example, organic acids can stimulate phosphate-solubilizing bacteria, while flavonoids enrich symbiotic and plant growth-promoting rhizobacteria [9]. This selective recruitment can lead to distinct microbial assemblages across different soil types. For example, *Arabidopsis thaliana* root exudates can specifically modulate microbial communities in nutrient-poor soils, with fast-growth-stage root exudates exhibiting higher functional potential and nutrient mobilization [48]. Additionally, root exudates can directly influence carbon and nitrogen cycling by priming the activity of soil organic matter degraders and inhibiting nitrification by nitrifying microorganisms [61]. Therefore, the observed differentiation in this study likely stems from inherent differences in native soil properties. Even when the same plant-derived signals are present, factors such as the initial microbial composition, soil adsorption characteristics, and physicochemical conditions (specifically, soil OM, DTN, pH, AP, AK, and EC) modulate the response of the microbiota to root exudates, ultimately leading to disparities in species-level composition.

The fungal communities were primarily made up of *Ascomycota* (80.27% in CL and 43.9% in SL), *Basidiomycota* (0.44% in CL and 3.74% in SL), and other taxa. The abundance of the top ten fungal phyla did not differ significantly between rhizosphere soils (CLR and SLR) and bulk soils (CLB and SLB) except *Mortierellomycota*, which was significantly (*p* < 0.05) enriched in SLR. A phylogenetic tree was constructed for the top 100 bacterial and fungal genera. As shown in Figure 2, the fungus *Fusarium* was absolutely dominant in terms of relative abundance, and *Penicillium* was the major fungal genus in the clay loam soil. The genus *Fusarium* contains many important plant pathogens and saprophytes, and its high abundance may be related to continuous tobacco cropping or soil background [62]. *Penicillium* is also a major fungal genus, playing an important role in soil ecological functions as a common source of saprophytic bacteria and some biocontrol bacteria [63]. Despite the overall convergence of rhizosphere community structure between the two soil types, soil-specific differences persisted at the OTU level, setting the stage for the core taxon identification in Section 3.4.

### 3.4. Core Rhizosphere-Adaptive Microbial Taxa Identification

In sandy loam soil, intensive planting has a greater regulatory effect on rhizosphere bacteria and fungi than in clay loam soil (Figure 3). Compared with bulk soil, OTUs enriched in the rhizosphere soil of both soil types were defined as the core rhizosphere-adaptive taxa. Volcano maps of different comparison groups in Figure 3 show that 38 bacterial OTUs were enriched in both CLR and SLR, with 35 OTUs depleted (Table S1). The number of differentially expressed fungal OTUs was higher in the SLR soils (27 enriched, 57 depleted) than in the CLR soils (2 enriched, 8 depleted). In contrast, no fungal OTUs were co-regulated in both soil types, indicating that the rhizosphere effect on the fungal community is soil-type-specific. Despite distinct initial microbial compositions, the same plant variety (K326) selected for similar rhizosphere microbial communities in different soils in terms of overall diversity and structure. However, significant soil-specific responses were retained at the level of individual taxa.

**Figure 4:**
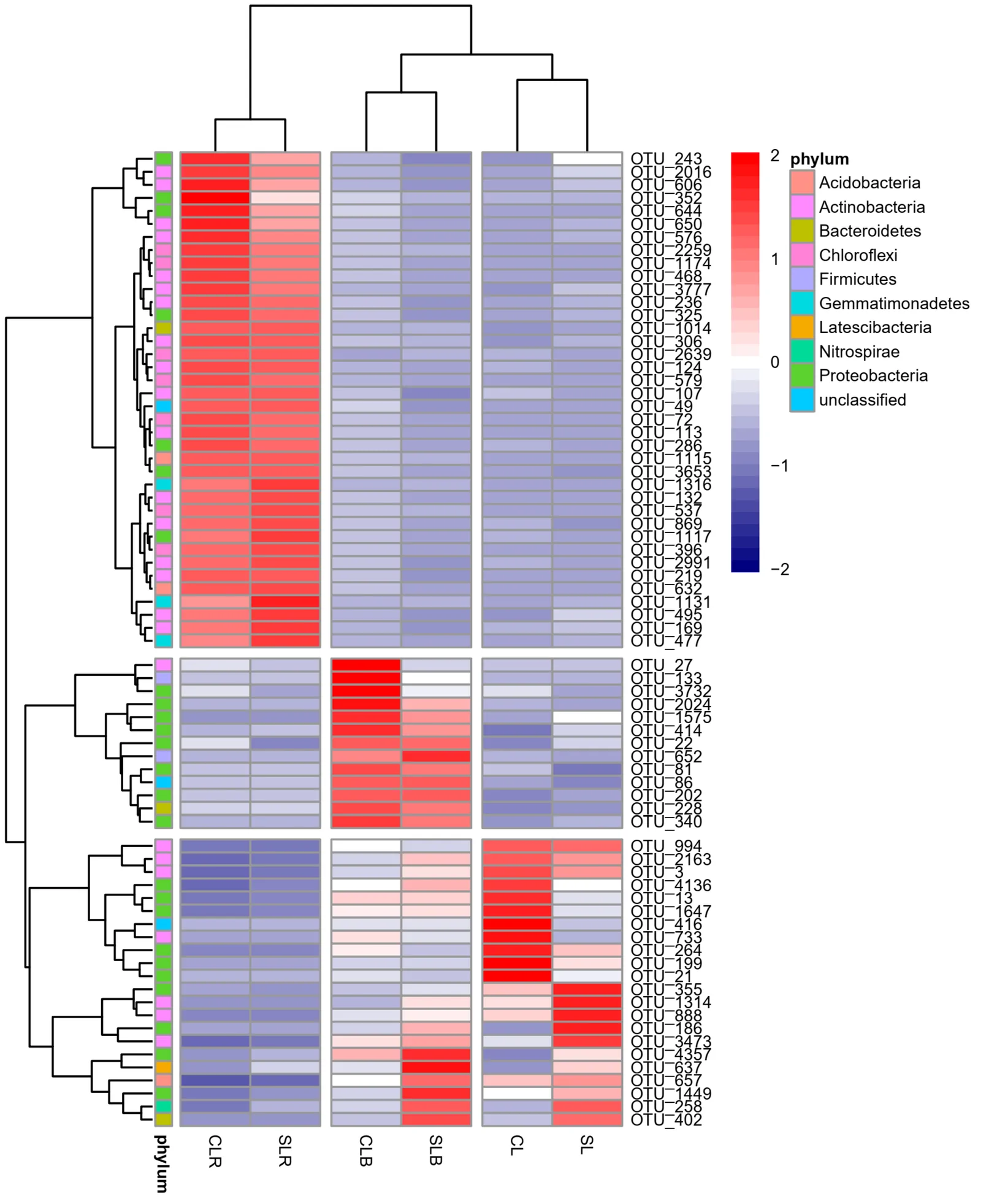
Heatmap of the bacterial OTUs enriched or depleted in the rhizosphere of the clay loam soil (CLR) and sandy loam soil (SLR). See Supplementary Table S1 for the complete taxonomic lineage of each OTU.

We further compared the bacterial OTUs that were simultaneously enriched and depleted in both CLR and SLR (Figure 4). The enriched OTUs were predominantly affiliated with *Actinobacteria* (especially *Acidimicrobiia* and *Thermoleophilia*), *Proteobacteria* and *Chloroflexi*, whereas depleted OTUs were mainly *Proteobacteria* (*Rhizobiales*) and *Actinobacteria* (especially *Micrococcales)* (Figure 4). We estimated enriched and depleted bacterial OTUs’ rrn copy numbers based on the rrnDB database. Intensive planting did not significantly change the bacterial growth rate and nutrient utilization efficiency (Figure S2). Among the bacteria enriched in the rhizosphere, K-strategists constituted the absolute majority (Table S2). We also calculated the niche breadth of the differentially abundant OTUs (Table S2). Among the rhizosphere-enriched bacteria, opportunists (broad niche breadth) represented the overwhelming majority (CLR-Up: 66.67%; SLR-Up: 86.4%), whereas specialists dominated among depleted taxa (CLR-Down: 67.15%; SLR-Down: 73.05%). This indicates that microbial taxa capable of rapidly responding to resource pulses in the rhizosphere gain a competitive advantage. These resource pulses may derive from multiple sources, including periodically released root exudates (e.g., sugars, organic acids, amino acids), nutrient inputs from fertilization, and the dynamic physicochemical gradients established by intensive cultivation. This likely implies that tobacco K326 is associated with the enrichment of a functionally similar bacterial core microbiome from distinct soil microbial pools. This exudate-sensitive microbiome is primarily composed of K-strategists that are efficient at utilizing resources in oligotrophic environments, and most of them are opportunists capable of rapidly exploiting nutrient pulses in the rhizosphere. This apparent plant-associated selection effect appears to partially override the differences inherent to the soil types.

The enriched core taxa exhibited a combination of genomic traits indicative of K-strategists (i.e., high resource efficiency) and ecological behaviors similar to opportunists (i.e., rapid response to exudates) (Table S2). This suggests they are “opportunistic K-strategists” or efficiency-focused generalists, whose genomic architecture is tuned for oligotrophy but whose expression allows them to capitalize on the rich, transient resources of the rhizosphere. Their strategy appears to be efficient resource collection and persistence in a competitive, high-carbon environment, not the establishment of extensive inter-species connections typical of network hubs [64]. The plant’s selective recruitment thus does not necessarily target microbial “keystone species” that govern network integrity [65]; instead, it may favor recruiting these functionally pre-adapted, resource-efficient opportunists that are highly proficient at utilizing root-derived carbon without integrating into the core network architecture [34,66]. The lack of significant change in their predicted growth and nutrient utilization functions (Figure S2) further confirms their pre-adaptation, requiring no major metabolic change upon enrichment.

In the CLR soil, eight fungal OTUs were depleted, identified as *Aspergillus candidus* (3), *Scopulariopsis koningii* (2), *Microascus brevicaulis* (1), *Fusicolla acetilerea* (1), and *Mortierella* sp. (1). Two fungal OTUs were enriched in the CLR soil, but neither could be identified to the species or genus level. In the SLR soils, 27 OTUs were enriched and 8 OTUs identified to the species level; they were identified as *Schizothecium inaequale* (2), *Fusarium delphinoides* (2), *Cercophora samala* (1), *Cladorrhinum bulbillosum* (1), *Gibberella baccata* (1), and *Talaromyces flavus* (1). A total of 57 OTUs were depleted and 12 OTUs identified to the species level; they were identified as *Zopfiella longicaudata* (5), *Phoma herbarum* (2), *Haptocillium balanoides* (2), *Cirrenalia iberica* (1), *Cladorrhinum brunnescens* (1), and *Loma morhua* (1). Among these differentially abundant OTUs, 11 enriched and 27 depleted OTUs belonged to the phylum Ascomycota. These differentially abundant fungal taxa provide a taxonomic foundation for the functional characterization of the rhizosphere microbiome presented in Section 3.5.

### 3.5. The Functions of the Core Rhizosphere-Adaptive Species and Microbial Community

To further clarify the roles of the enriched bacterial and fungal OTUs within the microbial co-occurrence networks of the two soil types, we performed Zi-Pi analysis. Co-occurrence network analysis revealed that the core rhizosphere-adapted bacterial OTUs predominantly occupied peripheral positions in both soil types (Figure S3), suggesting their limited role in maintaining network structural stability, which indicated that although intensive planting induced shifts in the bacterial communities of both soils, neither the enriched nor the depleted bacterial taxa played a significant role in maintaining the stability of the microbial network structure.

**Figure 5:**
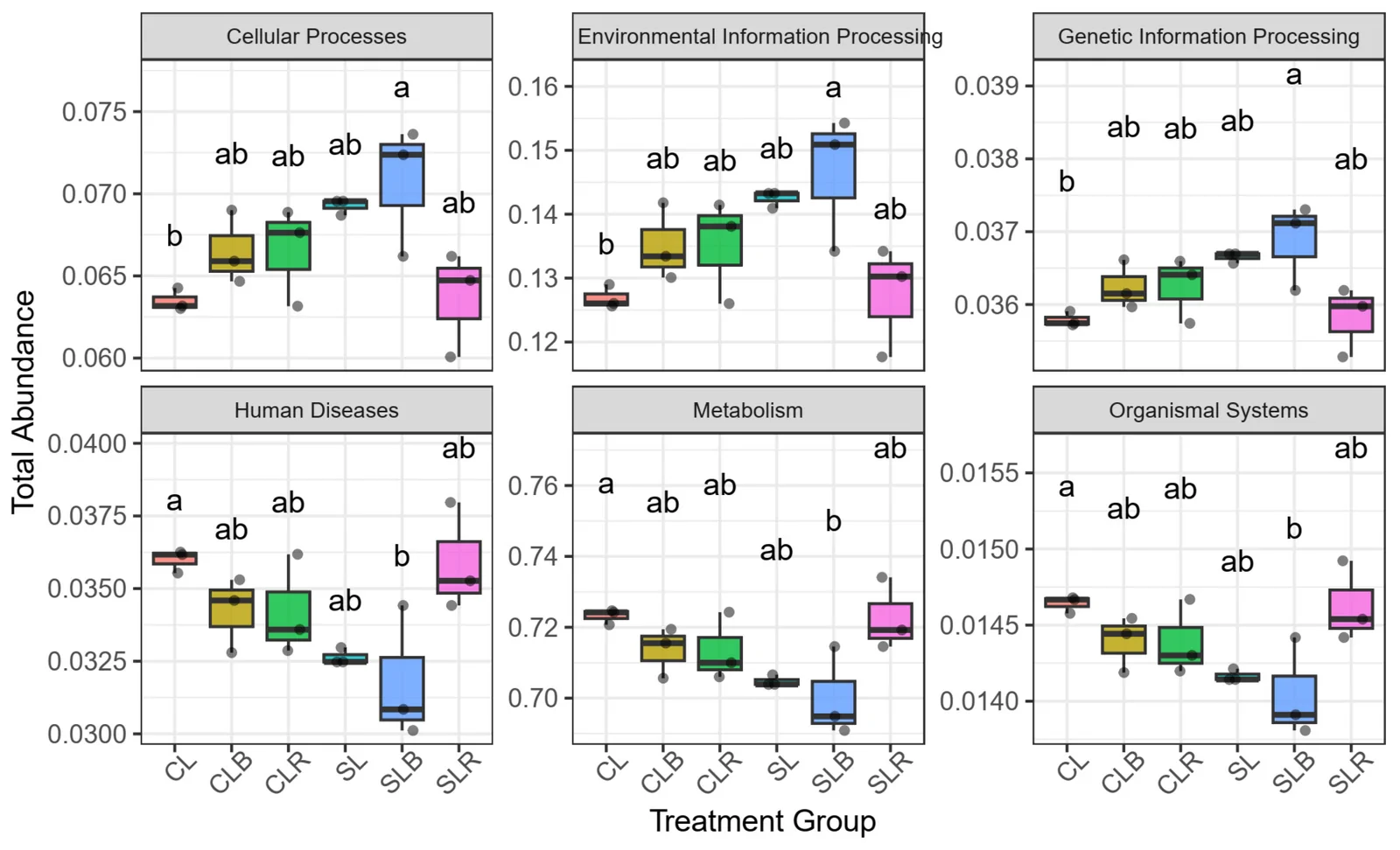
The metabolic functions of the 38 bacterial OTUs enriched in the rhizosphere of both soil types were predicted based on Tax4Fun2. Abbreviations: CL, clay loam soil; SL, sandy loam soil; B, bulk soil; R, rhizosphere soil. Different letters indicate significant differences based on one-way ANOVA followed by Tukey HSD test.

**Figure 6:**
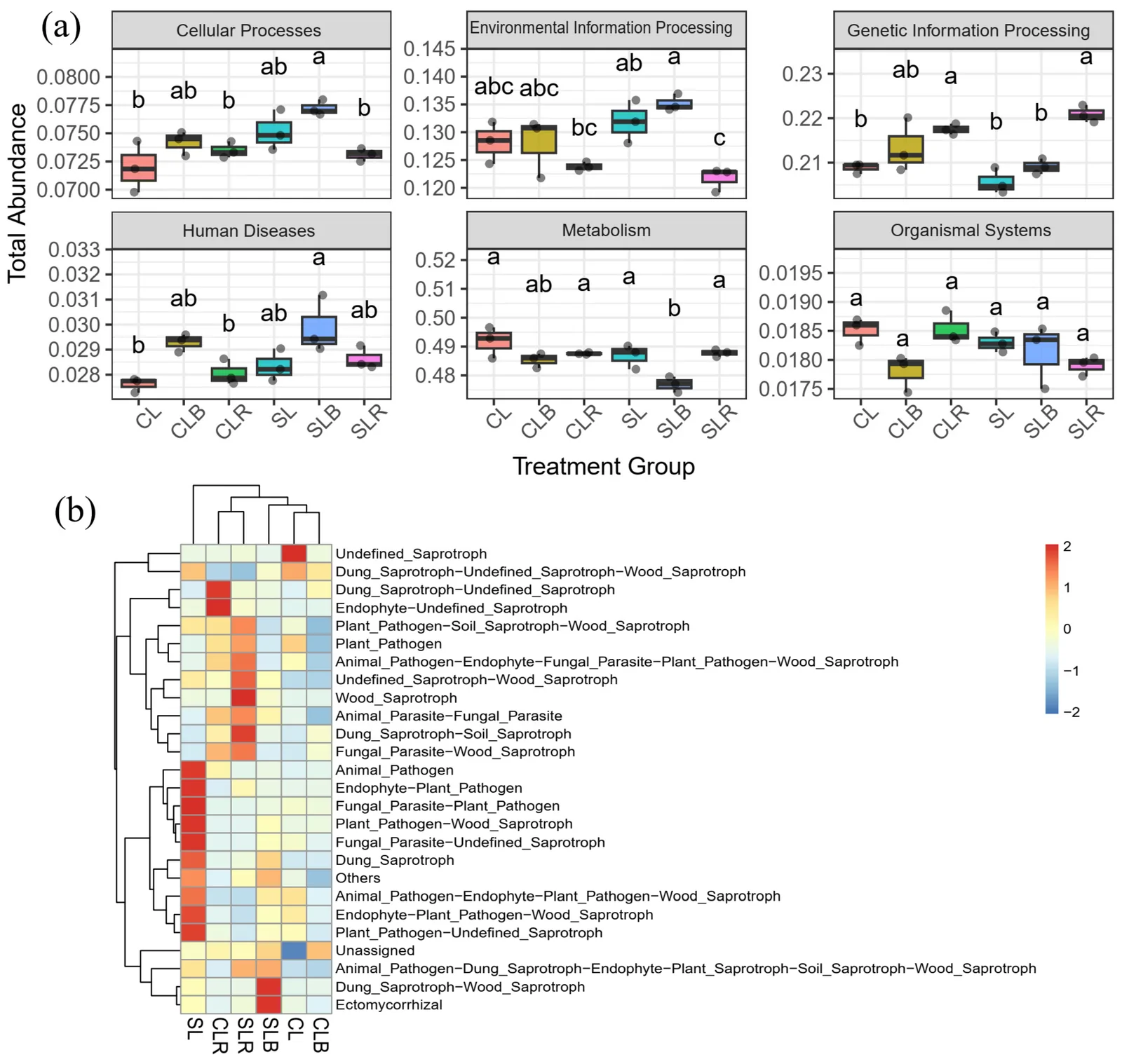
Comparisons of the predicted functions of the soil bacterial communities based on Tax4Fun2 (**a**) and fungal communities based on FunGuild (**b**). Abbreviations: CL, clay loam soil; SL, sandy loam soil; B, bulk soil; R, rhizosphere soil. Different letters in indicate significant differences based on one-way ANOVA followed by Tukey HSD test.

To further investigate the functions of the bacterial taxa enriched by intensive planting, we predicted the functional profiles of the 38 enriched bacterial OTUs using Tax4Fun2. The observation that the predicted functional profiles of these enriched bacterial OTUs remained largely unchanged (Figure 5) further supports the notion that soil microbial taxa are not functionally equivalent [18]. We speculate that these enriched core taxa (primarily Actinobacteria) may represent “opportunists” highly adept at utilizing root exudates and thriving in the high-carbon rhizosphere environment. Their functional profiles are probably inherently well-adapted to the rhizosphere niche, thus requiring no substantial functional shifts upon enrichment of some rhizosphere bacteria. This aligns with the concept that plants may recruit functionally specific taxa from the native soil microbial pool that are capable of performing conserved, critical functions (e.g., rapid carbon turnover) rather than generalists that influence broader community dynamics [7,67]. The significant functional shifts observed at the whole-community level in sandy loam soil (Figure 6) likely arise from the combined action of many taxa, including rare species, or from changes in gene expression rather than from the functional contribution of these enriched OTUs [68]. Future research employing isolation and cultivation of these enriched OTUs, coupled with direct functional validation, will be crucial to confirm their specific roles and understand the trade-offs involved in the plant’s microbiome management strategy.

### 3.6. The Effect of Soil Physicochemical Properties on the Microbial Communities of Different Soil Types

**Figure 7:**
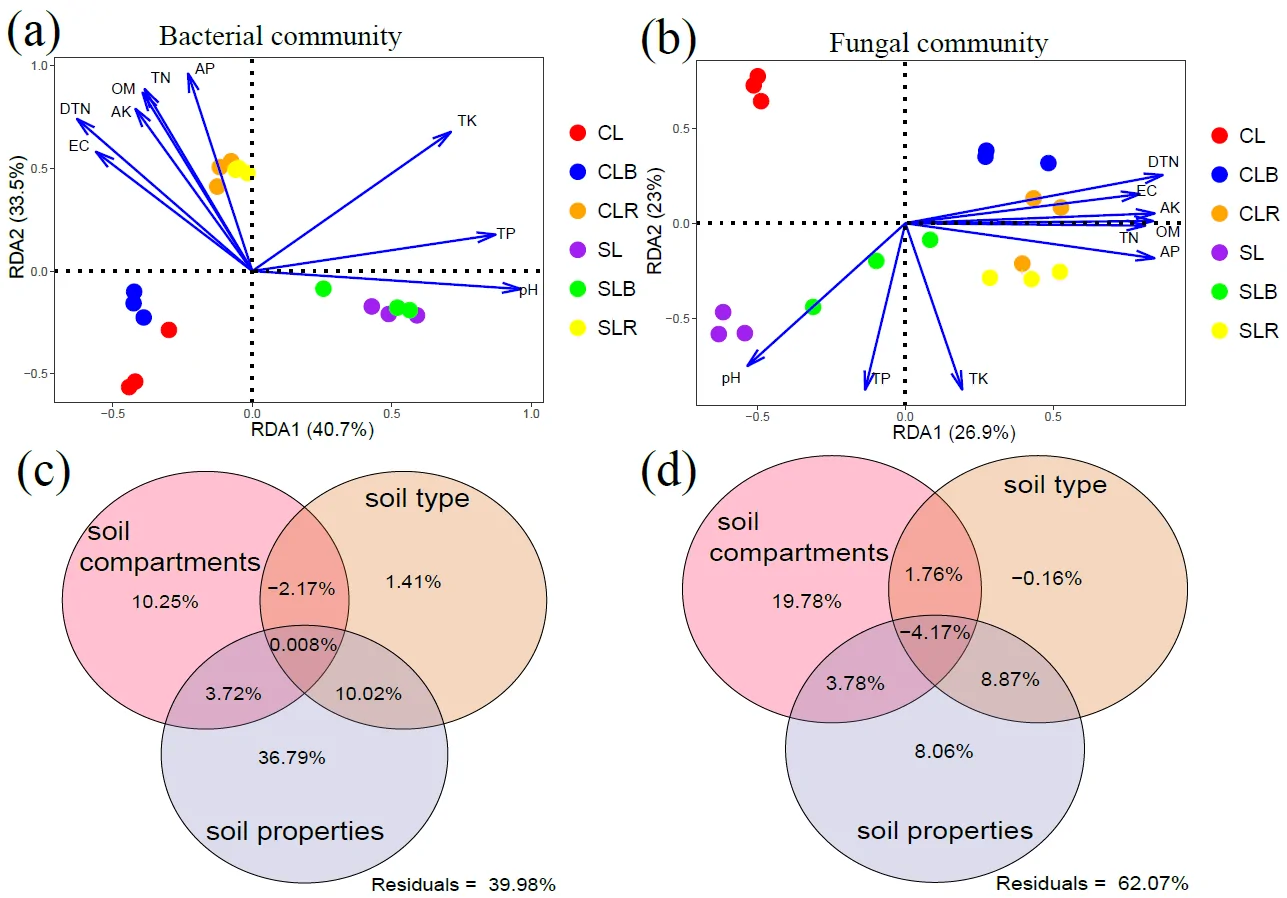
RDA scatter plots of the bacterial (**a**) and fungal (**b**) communities in clay loam (CL) or sandy loam (SL) soils planted with tobacco. Variation partitioning analysis showing the percentages of the variance in the bacterial (**c**) and fungal (**d**) communities explained by the soil physicochemical properties, soil type and soil compartments (i.e., bulk soil vs. rhizosphere soil). CLB and SLB represent bulk soil, while CLR and SLR represent rhizosphere soil. Environmental variables include OM, pH, TN, DTN, TP, AP, TK, AK, and EC. Abbreviations for soil physicochemical properties are shown in Table 1.

To further explore the associations between soil nutrients, soil type, and microbial community structure, we performed RDA and VPA analyses. RDA identified soil OM and DTN as the environmental variables most strongly correlated with both bacterial and fungal community structure (Figure 7), while VPA further revealed distinct assembly patterns between the two kingdoms. Soil physicochemical properties (e.g., OM, DTN) explained a larger proportion of bacterial community variation, whereas the soil compartment emerged as the dominant factor associated with fungal community structure (Figure 7). Specifically, the rhizosphere enrichment of OM (up to 54.56 g/kg in CLR and 47.74 g/kg in SLR) and DTN (up to 273.46 mg/kg in CLR and 227.37 mg/kg in SLR) coincided with a nutrient-rich microenvironment in which certain bacterial phyla (e.g., *Actinobacteria*, *Acidobacteria*, *Firmicutes*, *Gemmatimonadetes*) were preferentially enriched (Figure 1), consistent with the concept that bacteria function as “habitat specialists” whose composition is tightly coupled to local resource availability [69,70]. The prominence of OM and DTN as correlated variables suggests that they reflect the carbon and nitrogen resource spectrum associated with the rhizosphere niche. This resource-filtering effect was strong enough to partly overcome soil-type differences, leading to the convergence of core bacterial taxa (38 shared OTUs) across the two soils. In stark contrast, the fungal community demonstrates a greater dependence on the soil compartments. This is likely rooted in the ancient symbiotic relationship between fungi and plants [71]. Root exudates in the rhizosphere soil not only provide carbon substrates but also contain specific signaling molecules (e.g., strigolactones) that can directly and precisely recruit beneficial symbiotic fungi [72].

The observed convergence of the rhizosphere microbial communities (similar overall structure) across the two distinct soil types results from the strong plant-mediated selection and nutrient input overriding the native soil differences. The remaining divergence (differences in specific taxa) can be attributed to the modulation of this selection process by the initial soil microbial pool and inherent soil properties. A major limitation of this study is that the field experimental design cannot fully disentangle the effects of fertilization from those of plant roots. Consequently, the “rhizosphere effect” as defined here represents a combined effect of both factors. Future studies employing more precisely controlled pot or greenhouse experiments could help quantify the independent contributions and interactive effects of fertilization and root activity. Furthermore, integrating metabolomics approaches would be invaluable for elucidating the causal relationships between plant-derived signals and microbial responses [73]. We also acknowledge that our single-time-point sampling at the topping stage captures only a snapshot of the rhizosphere microbiome. Temporal dynamics from seedling to senescence may reveal additional shifts in community assembly and warrant investigation in future research.

## 4. Conclusions

Intensive planting created a strongly selective rhizosphere microenvironment with enriched nutrients (OM, DTN, AP, AK, TN) and pH converging toward the neutral–alkaline range (7.4–7.8). Bacteria and fungi responded differently to this selection. Bacterial diversity decreased slightly in the rhizosphere, and community assembly was driven by deterministic processes, forming a shared core microbiome of 38 OTUs across both soil types. In contrast, fungal diversity increased in the rhizosphere, but community structure remained soil-specific, with no shared core community, indicating that fungal assembly is more constrained by soil background than by plant selection. The 38 core bacterial OTUs (mainly from *Actinobacteria* and *Chloroflexi*) occupied peripheral positions in the co-occurrence network and showed no major functional shifts, suggesting that they are pre-adapted opportunists for resource use rather than keystone species for network stability. Bacterial communities were primarily shaped by soil OM and DTN, which acted as the main environmental filters. Fungal communities appeared to be more influenced by the soil compartment, reflecting a closer symbiotic relationship with the plant. In summary, soil type serves as an indispensable “meta-filter” that, together with agricultural management, determines rhizosphere microbial assembly. This study provides insights into plant–microbe–soil interactions and offers a theoretical basis for microbiome-based soil management.

## Figures and Tables

**Table 1:** Physicochemical properties of bulk and rhizosphere clay loam (CL) and sandy loam (SL) soils under intensive tobacco cultivation.

	SL	SLR	SLB	CL	CLR	CLB
OM (g/kg)	3.2 ± 0.6 c	47.7 ± 5.5 a	3.6 ± 0.7 c	11.5 ± 0.8 bc	54.6 ± 11.5 a	20.6 ± 2.3 b
DTN (mg/kg)	28.5 ± 2.9 d	227.4 ± 12.5 ab	35.3 ± 7.7 d	112.2 ± 9.2 c	273.5 ± 37.6 a	180.6 ± 14.3 b
AP (mg/kg)	13.8 ± 0.1 c	82.3 ± 2.2 a	15.8 ± 0.9 c	13.0 ± 1.0 c	85.9 ± 2.2 a	29.2 ± 1.4 b
AK (mg/kg)	37.5 ± 2.5 c	359.2 ± 57.8 ab	34.2 ± 1.4 c	67.5 ± 15.2 c	542.5 ± 34.4 a	296.7 ± 179.2 b
TN (g/kg)	0.22 ± 0.04 c	2.79 ± 0.5 a	0.22 ± 0.05 c	0.72 ± 0.03 bc	2.93 ± 0.24 a	1.08 ± 0.08 b
TP (g/kg)	6.83 ± 2.06 a	6.7 ± 0.62 a	8.24 ± 0.03 a	0.92 ± 0.12 b	2.33 ± 0.85 b	1.49 ± 0.35 b
TK (g/kg)	14.96 ± 0.2 a	16.24 ± 0.32 a	14.85 ± 0.33 a	7.13 ± 0.37 b	15.8 ± 0.28 a	6.85 ± 1.38 b
pH	8.75 ± 0.14 a	7.83 ± 0.1 b	8.8 ± 0.2 a	7.39 ± 0.28 b	7.41 ± 0.08 b	6.78 ± 0.21 c
EC (μS/cm)	67.6 ± 11.4 c	326.7 ± 89.1 a	76.1 ± 9.1 c	111.5 ± 38.3 bc	246.7 ± 36.7 ab	327 ± 105.2 a

Abbreviations: CL, clay loam soil; SL, sandy loam soil; CLB, clay loam bulk soil; CLR, clay loam rhizosphere soil; SLB, sandy loam bulk soil; SLR, sandy loam rhizosphere soil. EC, electrical conductivity; OM, organic matter; TN, total nitrogen; DTN, dissolved total nitrogen; TP, total phosphorus; AP, available phosphorus; TK, total potassium; AK, available potassium. Note: Data are presented as mean ± standard deviation (SD) with n = 3 biological replicates per treatment. Different letters indicate significant differences based on one-way ANOVA followed by a Duncan test.

**Table 2:** The diversity of the bacterial and fungal communities in tobacco rhizosphere and bulk soils.

	Treatment	Observed Species	Diversity Index ^1^		Richness Index		Coverage ^2^
			Shannon	Simpson	Chao1	ACE	
Bacteria	CL	1639 ± 163.04 b	8.21 ± 0.29 b	0.987 ± 0.004 a	1890.45 ± 231.22 b	1974.7 ± 229.71 b	0.982 ± 0.003 a
	CLB	2039.67 ± 190.35 a	9.1 ± 0.16 a	0.994 ± 0.001 a	2389.53 ± 303.14 ab	2466.93 ± 303.37 ab	0.977 ± 0.005 a
	CLR	1969 ± 70.93 ab	9.02 ± 0.47 a	0.987 ± 0.014 a	2235.01 ± 138.21 ab	2294.29 ± 148.24 ab	0.981 ± 0.003 a
	SL	1946.67 ± 101.16 ab	9.1 ± 0.22 a	0.994 ± 0.002 a	2309.19 ± 94.69 ab	2383 ± 61.25 ab	0.978 ± 0.002 a
	SLB	2225.67 ± 177.07 a	9.51 ± 0.14 a	0.996 ± 0.001 a	2559.54 ± 221.96 a	2622.71 ± 255.2 a	0.977 ± 0.003 a
	SLR	2066.33 ± 80.16 a	9.42 ± 0.07 a	0.996 ± 0 a	2468.83 ± 215.67 ab	2439.04 ± 175.2 ab	0.979 ± 0.004 a
Fungi	CL	818 ± 151.25 a	5.94 ± 0.64 a	0.933 ± 0.029 a	979.94 ± 146.88 a	1001.21 ± 133.63 a	0.994 ± 0.001 a
	CLB	744 ± 273.65 a	5.63 ± 1.29 a	0.921 ± 0.051 a	978.66 ± 325.31 a	954.94 ± 301.23 a	0.994 ± 0.002 a
	CLR	1013 ± 170.24 a	6.71 ± 0.79 a	0.96 ± 0.027 a	1255.94 ± 225.06 a	1274.34 ± 225.09 a	0.992 ± 0.002 a
	SL	1109.67 ± 102.5 a	7.38 ± 0.53 a	0.979 ± 0.009 a	1241.83 ± 160.1 a	1244.84 ± 149.14 a	0.995 ± 0.003 a
	SLB	1020 ± 303.24 a	6.99 ± 0.88 a	0.973 ± 0.018 a	1207.64 ± 282.44 a	1214.18 ± 275.85 a	0.993 ± 0.001 a
	SLR	1165 ± 93.54 a	7.22 ± 0.27 a	0.976 ± 0.008 a	1427.17 ± 123.8 a	1465.09 ± 114.19 a	0.991 ± 0.001 a

Note: Different lowercase letters after data represent significant differences at the *p* < 0.05 level, as tested by Tukey’s test. ^1^: The higher the Shannon index and the lower the Simpson index, the higher the diversity of bacterial communities. ^2^: Coverage: The higher the value, the lower the probability of unmeasured sequences in the sample. Abbreviations: CL, clay loam soil; SL, sandy loam soil; B, bulk soil; R, rhizosphere soil.

## Data Availability

The data presented in this study are openly available in the NCBI Sequence Read Archive (SRA) database at [https://www.ncbi.nlm.nih.gov/] [PRJNA1481388].

## Supplementary Materials

The following supporting information can be downloaded at https://www.mdpi.com/article/10.3390/microorganisms14081849/s1. Figure S1: Comparative Venn diagrams illustrating the overlap among three experimental groups in clay loam (CL) or sandy loam (SL) soils planted with tobacco;Figure S2: Average rrn copy numbers of bacterial OTUs in the soils; Figure S3: Network roles (Zi-Pi plot) revealing the keystone species of the microbial community. Table S1: Correspondence between OTU IDs and taxonomic assignments for the bacterial OTUs both enriched or depleted in the rhizosphere of both soil types (CLR and SLR); Table S2: Number of ribosomal RNA operons (rrn) and niche breadth of the enriched and depleted bacteria OTUs.
