# Supplementary material for: Convergent Bacterial but Divergent Fungal Communities in the Tobacco Rhizosphere Under Intensive Management on Contrasting Soils
Source: Microorganisms. 2026 Aug 20;14(8):1849. doi: 10.3390/microorganisms14081849 (PMC13515114; doi:10.3390/microorganisms14081849)
Supplement: Supplementary file 1 [file microorganisms-14-01849-s001.zip › microorganisms-4460919-supplementary.pdf]

Table S1. Correspondence between OTU IDs and taxonomic assignments for the bacterial OTUs both enriched or depleted in the rhizosphere of both soil types (CLR and SLR)

OTU number	Differential types	Phylum	Class	Order	Family	Genus	Species
OTU_13	depleted	Proteobacteria	Alphaproteobacteria	Rhizobiales	Xanthobacteraceae	Bradyrhizobium	Bradyrhizobium_elkanii
OTU_1314	depleted	Actinobacteria	unidentified_Actinobacteria	Propionibacteriales	Nocardioidaceae	Nocardioides	unclassified
OTU_133	depleted	Firmicutes	Bacilli	Bacillales	Bacillaceae	Bacillus	Bacillus_galactosidilyticus
OTU_1449	depleted	Proteobacteria	Gammaproteobacteria	unidentified_Gammaproteobacteria	unidentified_Gammaproteobacteria	Acidibacter	unclassified
OTU_1575	depleted	Proteobacteria	Alphaproteobacteria	Sphingomonadales	Sphingomonadaceae	unclassified	unclassified
OTU_1647	depleted	Proteobacteria	Alphaproteobacteria	Rhizobiales	Xanthobacteraceae	Bradyrhizobium	unclassified
OTU_186	depleted	Proteobacteria	Gammaproteobacteria	Xanthomonadales	Xanthomonadaceae	Lysobacter	unclassified
OTU_199	depleted	Proteobacteria	Gammaproteobacteria	unidentified_Gammaproteobacteria	Burkholderiaceae	unclassified	unclassified
OTU_202	depleted	Proteobacteria	Alphaproteobacteria	Rhizobiales	unidentified_Rhizobiales	Devosia	unclassified
OTU_2024	depleted	Proteobacteria	Gammaproteobacteria	Pseudomonadales	Moraxellaceae	Acinetobacter	unclassified
OTU_21	depleted	Proteobacteria	Gammaproteobacteria	unidentified_Gammaproteobacteria	Burkholderiaceae	Ramlibacter	unclassified
OTU_2163	depleted	Actinobacteria	unidentified_Actinobacteria	Micrococcales	Micrococcaceae	Pseudarthrobacter	unclassified
OTU_22	depleted	Proteobacteria	Gammaproteobacteria	unidentified_Gammaproteobacteria	Burkholderiaceae	Pseudoduganella	unclassified
OTU_228	depleted	Bacteroidetes	Bacteroidia	Cytophagales	Spirosomaceae	Dyadobacter	unclassified
OTU_258	depleted	Nitrospirae	Nitrospira	Nitrospirales	Nitrospiraceae	unidentified_Nitros	Nitrospira_sp

						piraceae	
OTU_264	depleted	Proteobacteria	Gammaproteobacteria	unidentified_Gammaproteobacteria	Nitrosomonadaceae	unidentified_Nitrosomonadaceae	unclassified
OTU_27	depleted	Actinobacteria	unidentified_Actinobacteria	Micrococcales	Brevibacteriaceae	Brevibacterium	Brevibacterium_epidermidis
OTU_3	depleted	Actinobacteria	unidentified_Actinobacteria	Micrococcales	Micrococcaceae	Arthrobacter	unclassified
OTU_340	depleted	Proteobacteria	Alphaproteobacteria	Caulobacterales	Caulobacteraceae	Brevundimonas	Brevundimonas_bullata
OTU_3473	depleted	Actinobacteria	unidentified_Actinobacteria	Propionibacteriales	Nocardioidaceae	Nocardioides	Nocardioides_simplex
OTU_355	depleted	Proteobacteria	Gammaproteobacteria	unidentified_Gammaproteobacteria	Nitrosomonadaceae	unclassified	unclassified
OTU_3732	depleted	Proteobacteria	Gammaproteobacteria	unidentified_Gammaproteobacteria	Burkholderiaceae	unclassified	unclassified
OTU_402	depleted	Bacteroidetes	Bacteroidia	Chitinophagales	Chitinophagaceae	unclassified	unclassified
OTU_4136	depleted	Proteobacteria	Alphaproteobacteria	Rhizobiales	Xanthobacteraceae	unclassified	unclassified
OTU_414	depleted	Proteobacteria	Alphaproteobacteria	Rhizobiales	unidentified_Rhizobiales	Devosia	unclassified
OTU_416	depleted	unclassified	unclassified	unclassified	unclassified	unclassified	unclassified
OTU_4357	depleted	Proteobacteria	Alphaproteobacteria	Rhizobiales	Xanthobacteraceae	Pseudolabrys	unclassified
OTU_637	depleted	Latescibacteria	unclassified	unclassified	unclassified	unclassified	unclassified
OTU_652	depleted	Firmicutes	Bacilli	Bacillales	Bacillaceae	Oceanobacillus	Oceanobacillus_caeni
OTU_657	depleted	Acidobacteria	Acidobacteriia	Solibacterales	unidentified_Solibacterales	Bryobacter	unclassified
OTU_733	depleted	Actinobacteria	Thermoleophilia	Solirubrobacterales	unclassified	unclassified	unclassified

OTU_81	depleted	Proteobacteria	Alphaproteobacteria	Caulobacterales	Caulobacteraceae	unclassified	unclassified
OTU_86	depleted	unclassified	unclassified	unclassified	unclassified	unclassified	unclassified
OTU_888	depleted	Actinobacteria	Acidimicrobiia	unclassified	unclassified	unclassified	unclassified
OTU_994	depleted	Actinobacteria	unidentified_Actinobacteria	Frankiales	unclassified	unclassified	unclassified
OTU_1014	enriched	Bacteroidetes	Bacteroidia	Chitinophagales	Chitinophagaceae	Terrimonas	unclassified
OTU_107	enriched	Actinobacteria	unclassified	unclassified	unclassified	unclassified	unclassified
OTU_1115	enriched	Acidobacteria	unclassified	unclassified	unclassified	unclassified	unclassified
OTU_1117	enriched	Proteobacteria	Alphaproteobacteria	unidentified_Alphaproteobacteria	Geminicoccaceae	unclassified	unclassified
OTU_113	enriched	Actinobacteria	unidentified_Actinobacteria	Propionibacteriales	Propionibacteriaceae	unclassified	unclassified
OTU_1131	enriched	Gemmatimonadetes	unidentified_Gemmatimonadetes	Gemmatimonadales	Gemmatimonadaceae	unclassified	unclassified
OTU_1174	enriched	Chloroflexi	Anaerolineae	Caldilineales	Caldilineaceae	Litorilinea	unclassified
OTU_124	enriched	Actinobacteria	Thermoleophilia	Gaiellales	unclassified	unclassified	unclassified
OTU_1316	enriched	Gemmatimonadetes	unidentified_Gemmatimonadetes	Gemmatimonadales	Gemmatimonadaceae	unclassified	unclassified
OTU_132	enriched	Actinobacteria	unidentified_Actinobacteria	unidentified_Actinobacteria	unidentified_Actinobacteria	unidentified_Actinobacteria	Actinobacteria_bacterium_RBG_19FT_COMBO_70_19
OTU_169	enriched	Actinobacteria	Thermoleophilia	Solirubrobacterales	unclassified	unclassified	unclassified
OTU_2016	enriched	Actinobacteria	Thermoleophilia	Gaiellales	unclassified	unclassified	unclassified
OTU_219	enriched	Actinobacteria	Acidimicrobiia	unclassified	unclassified	unclassified	unclassified

OTU_2259	enriched	Chloroflexi	Dehalococcoidia	unidentified_Dehalococcoidia	unidentified_Dehalococcoidia	unidentified_Dehalococcoidia	bacterium_YC-LK-LKJ27
OTU_236	enriched	Actinobacteria	Thermoleophilia	Gaiellales	unclassified	unclassified	unclassified
OTU_243	enriched	Proteobacteria	Alphaproteobacteria	Rhodospirillales	unidentified_Rhodospirillales	Defluviicoccus	unclassified
OTU_2639	enriched	Chloroflexi	unclassified	unclassified	unclassified	unclassified	unclassified
OTU_286	enriched	Proteobacteria	Alphaproteobacteria	Rhodobacterales	Rhodobacteraceae	Amaricoccus	unclassified
OTU_2991	enriched	Actinobacteria	unidentified_Actinobacteria	Propionibacteriales	Nocardioidaceae	unclassified	unclassified
OTU_306	enriched	Actinobacteria	Acidimicrobiia	unclassified	unclassified	unclassified	unclassified
OTU_325	enriched	Proteobacteria	Gammaproteobacteria	unidentified_Gammaproteobacteria	Nitrosomonadaceae	unclassified	unclassified
OTU_352	enriched	Proteobacteria	Deltaproteobacteria	Myxococcales	Archangiaceae	Anaeromyxobacter	Anaeromyxobacter_sp_Fw109-5
OTU_3653	enriched	Proteobacteria	Alphaproteobacteria	Rhizobiales	unidentified_Rhizobiales	unclassified	unclassified
OTU_3777	enriched	Actinobacteria	Thermoleophilia	Gaiellales	Gaiellaceae	Gaiella	unclassified
OTU_396	enriched	Chloroflexi	Chloroflexia	Thermomicrobiales	unclassified	unclassified	unclassified
OTU_468	enriched	Actinobacteria	Acidimicrobiia	Actinomarinales	unclassified	unclassified	unclassified
OTU_477	enriched	Gemmatimonadetes	unidentified_Gemmatimonadetes	Gemmatimonadales	Gemmatimonadaceae	unclassified	unclassified
OTU_49	enriched	unclassified	unclassified	unclassified	unclassified	unclassified	unclassified
OTU_495	enriched	Actinobacteria	Thermoleophilia	Solirubrobacterales	unclassified	unclassified	unclassified
OTU_537	enriched	Chloroflexi	unclassified	unclassified	unclassified	unclassified	unclassified
OTU_576	enriched	Actinobacteria	Thermoleophilia	Gaiellales	Gaiellaceae	Gaiella	unclassified

OTU_579	enriched	Chloroflexi	Anaerolineae	unclassified	unclassified	unclassified	unclassified
OTU_606	enriched	Actinobacteria	Acidimicrobiia	unclassified	unclassified	unclassified	unclassified
OTU_632	enriched	Acidobacteria	unclassified	unclassified	unclassified	unclassified	unclassified
OTU_644	enriched	Proteobacteria	Alphaproteobacteria	unidentified_Alphaprot eobacteria	Geminicoccaceae	unclassified	unclassified
OTU_650	enriched	Actinobacteria	Acidimicrobiia	unclassified	unclassified	unclassified	unclassified
OTU_72	enriched	Chloroflexi	unclassified	unclassified	unclassified	unclassified	unclassified
OTU_869	enriched	Actinobacteria	Acidimicrobiia	unclassified	unclassified	unclassified	unclassified

Table S2 Number of ribosomal RNA operons (rrn) and niche breadth of the enriched and depleted bacteria OTUs

	OTU number	Number of ribosomal RNA operons[a]			Niche breadth		
		rrn≤2	2<rrn<5	rrn≥5	generalists	specialists	opportunists
CLR-Up	66	42 (63.6%)	13 (19.7%)	11 (16.7%)	0	22 (33.33%)	44 (66.67%)
CLR-Down	417	221 (53.0%)	151 (36.2%)	45 (10.8%)	3 (0.72%)	280 (67.15%)	134 (32.13%)
SLR-Up	250	178 (71.2%)	49 (19.6%)	23 (9.2%)	0	34 (13.6%)	216 (86.4%)
SLR-Down	423	264 (62.4%)	141 (33.3%)	18 (4.3%)	0	309 (73.05%)	114 (26.95%)
Enriched OTUs in CLR and SLR	38	31 (81.6%)	6 (15.8%)	1 (2.6%)	0	9 (23.68%)	29 (76.32%)
Depleted OTUs in CLR and SLR	35	17 (48.6%)	14 (40.0%)	4 (11.4%)	0	25 (71.42%)	10 (28.57%)

Note: a: Bacterial communities with fewer rrn operons tend to adopt a K-strategy, while those possessing more rrn operons tend to follow an r-strategy.
Abbreviations: CL, clay loam soil; SL, sandy loam soil; B, bulk soil; R, rhizosphere soil.

Figure S1 Venn diagrams

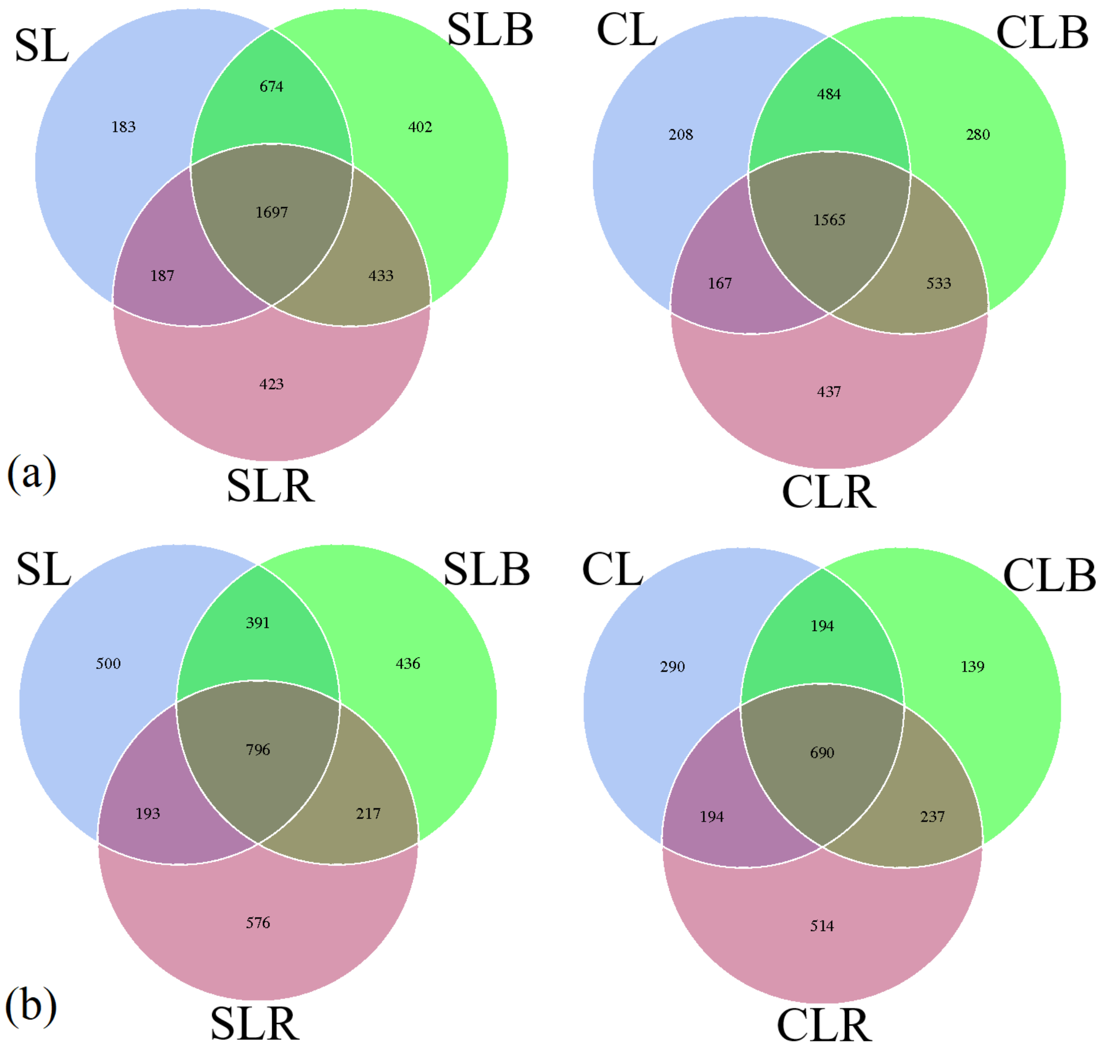

Figure S1 Comparative Venn diagrams illustrating the overlap among three experimental groups in clay loam (CL) or sandy loam (SL) soils planted with tobacco. CLB and SLB represent bulk soil, while CLR and SLR represent tobacco rhizosphere soil.

Figure S2 Average rrn copy numbers

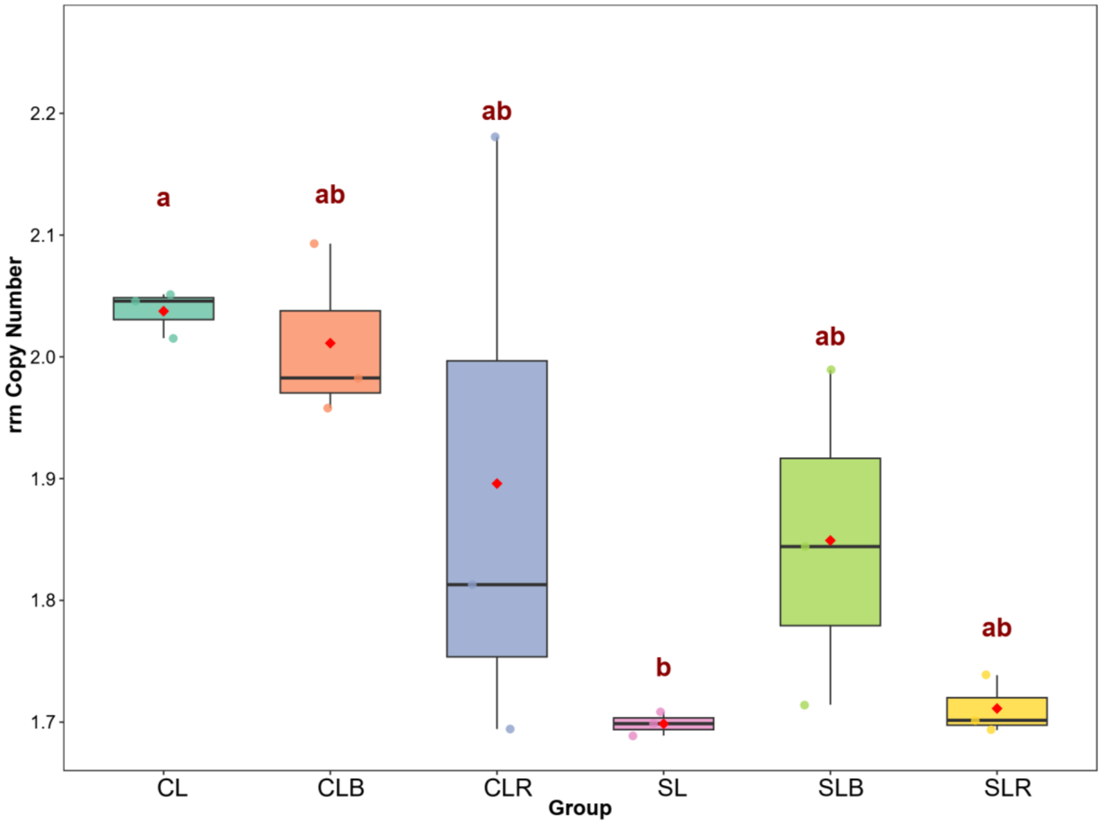

Figure S2 Average rrn copy numbers of bacterial OTUs in the soils. The boxs indicate mean values and the error bars indicate standard errors. Unadjusted P values of one-way ANOVA followed by Tukey HSD test are labeled as letters, different letters indicate significant differences ($p < 0.05$).

Figure S3 Zi-Pi plot

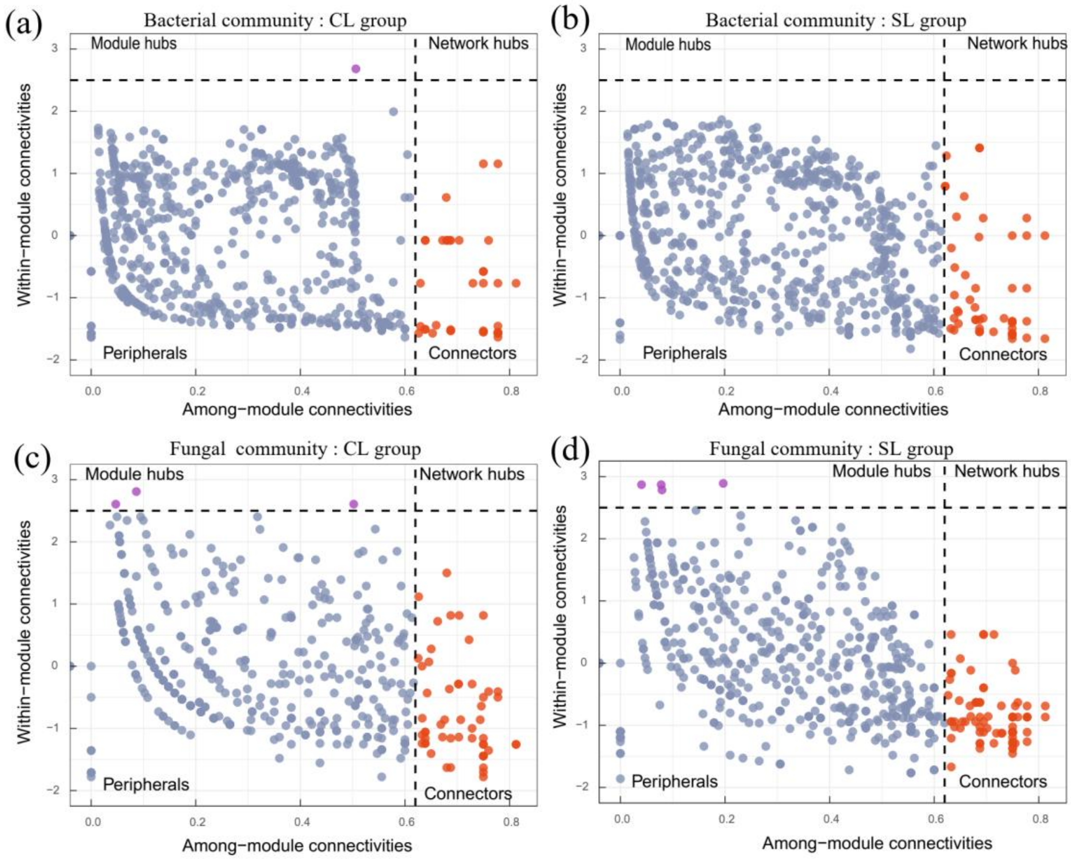

Figure S3 Network roles (Zi-Pi plot) revealing the keystone species of the microbial community. CL, clay loam soil; SL, sandy loam soil.
